# Introductory Address to the Sixty-Fifth Annual Session of the Medical Department of the University of Buffalo

**Published:** 1910-11

**Authors:** Thew Wright

**Affiliations:** Buffalo, N. Y. 152 Allen Street


### Introductory Address to the Sixty-fifth Annual Session of the Medical Department of the University of Buffalo

By THEW WRIGHT, M.D.
Buffalo, N. Y.

IT is my privilege to welcome you to these halls of learning and in so doing to open the sixty-fifth annual session of this school. An occasion such as this seems to me one of more than ordinary seriousness. Until now, study for most of you has been but an incident in your lives. I venture to say but few of you have given serious thought to your high school work. There were certain tasks set to be performed and accepted as inevitable and executed. Much of your work has been of such a character that you were galled by the feeling that your labor was being wasted upon such mental gymnastics. But from now on your attitude toward study is different.

For most of you in fact and for the rest of you in fancy at least, the die is cast and you are now entering upon the course of study that is to definitely prepare you for your life's work. As in most things so here the way you start to a great extent determines your success or failure. As in a footrace a false start may handicap the runner beyond his power to recover, so in the study of medicine unless one starts with the proper ideas and methods of study true success cannot be attained.

Now what is the first essential of success? It is the same here as in other walks of life,—character. No one has ever been truly successful without character as a foundation. Nowhere is that truer than in our profession; and do not count successful those who, considering the practice of medicine a business and applying to it the methods of the modern business world and trafficing in the lives and health of those entrusted to their care, have gained some wealth and prominence.

If success is to be measured by the dollars gained then our claim that the profession of medicine stands on a higher plane than business and is more to be respected and honored, is a false one.

But that claim is not false. In no other walk of life, except it be the ministry, do men give their services for no money gain as they do in ours. In no other are those who conscientiously do their work called upon continually to do things for the good of others at the expense of their own welfare. No other profession can glory in the fact that much of its effort is spent in diminishing the need of it.

The man who corners the wheat market is considered smart in the business world, but what would you think of the doctor who cornered the supply of antitoxin in a diphtheria epidemic, or who failed to quarantine his patients that he might have more cases to treat?

The fact that there are some in our ranks whose ideals are not those of the true physician should not condemn the profession as a whole. The curse of the present age is the spirit of commercialism, and that it has tainted our profession is not to be wondered at. But the conscience of the profession has been awakened and steps are being taken to root out this evil.

You are here to fit yourselves to enter a profession than which there is none more honorable, none which demands greater self-sacrifice, more self-restraint, or in short, more character. From the moment a man sets his course to become a physician he should be imbued with the sense of the responsibility which he has voluntarily assumed. He has chosen a calling in which he will be confronted daily with situations whereupon his good judgment will depend life or death, health or days of misery to some of his fellowmen. And not that alone, for he is preparing for a profession which will bring him closer to the souls of his fellow men than any other, not excepting the clergy. It will often fall to the lot of each of you to throw your influence upon the side of right and to be of help to those suffering from moral as well as physical ills. The physician knows the innermost soul of his patient ofttimes when it is hidden from everyone else.

The family doctor's knowledge of anatomy is not limited to that learned in the medical school, but includes an array of skeletons from the closets of his patients far more ghastly than those whose acquaintance you will make in the anatomic laboratory of this college. The family doctor of fifty years ago who was friend and adviser in many ways other than medical, has passed with the times, at least in our larger cities. But the time will never come until human nature changes, when the physician shall cease to stand in the closest relationship to his patient and to be called upon for help and advice.

You are preparing for a profession where demands upon your physical, your mental, and your moral strength exceed that of any other walk of life. You will be confronted with work that will rob you of your rest until it will seem as though the limit of endurance had been reached, and the words of Fernel so fittingly placed in our surgical clinic, "Destiny reserves for us repose enough," a hollow mockery. You will be confronted with problems that so tax your brain as to make you feel you were worse than a fool to have ever entered the profession of medicine. You will be placed in positions of temptation where the wrong course seems so easy and alluring that more than human power is needed to resist it. This has been the experience of all who have followed our profession and is a part of it. And for such trials you must be preparing yourselves, as well as to learn to diagnosticate your patients' bodily ills and what to do for them.

Your obligations to your future profession begins here and not when you leave this school the proud possessor of the coveted M.D. degree. Many of you are for the first time free from the restraint of home influences and whether willing to admit it or not, have a feeling of independence and of now being a man, that is all too fertile soil for the many temptations that assail one at this time. But in four short years you will be entrusted with the lives and health of your fellowmen. Does not the time seem short? Does that not give you somewhat a feeling of awe?

You will get from your college course just what you put into it. Nowhere is it truer that "as ye sow so shall ye reap." It is only by hard and conscientious work that you can get from your course all that is offered you, and what you have a right to expect. What is this medical course? It is but the beginning of a life of study. I trust that none of you look upon the study of medicine as upon the learning of a trade; that none of you feel that you are here to study four years after which you will have learned your trade and are fitted to go and practise it, as the joiner's apprentice serves his time and then has learned all there is to know about his work and is a master joiner.

You have a right to expect from your four years here a solid foundation in the principles of medicine. You have a right to expect that you will be well grounded in those subjects essential to the proper understanding of disease and our present means of combating and preventing it, which must be common to all branches of the profession. That much you can acquire and more.

The problem of medical education has become a most complex one. The advances made in medicine and surgery during the past fifty years have been as great as those in other lines of science. Fifty years ago the college course consisted of two terms of lectures, the second term being but a repetition of the first. When this school was founded it was possible for one man to know nearly all that was known of the practice of medicine and surgery. Today for a man to be the master of any one branch places him among those whom we call great. To but catalogue the advances and discoveries made during the past half century would consume more time than I have at my disposal on this occasion. But with this great advance in the science of medicine there is danger of slighting the art. There is today a tendency that must be guarded against of overestimating the value of the purely scientific and laboratory side of medicine to the practical man.

The business of a school like this is to make useful working physicians and to succeed in this it is almost as important not to overcrowd the mind of the pupil with merely curious knowledge, as it is to store it with useful information. There will be much during your college course that may seem to you unessential and of no practical application. But all systematic knowledge involves much that is not practical; it is, however, the only kind of knowledge that is satisfactory, and systematic study proves, in the long run, the easiest way of acquiring and retaining facts that are practical.

I urge you to study the subjects assigned to you with all vigor. I warn you of the mistake that many of us have made, of trying as undergraduates to estimate the relative importance of the different studies we were called upon to pursue. The course of study prepared for you is not a conglomeration of subjects chosen at random, but is a carefully and thoughtfully planned and selected group, each member of which is of importance in itself and in its relation to the others. There may be many things that in after years you can afford to forget, which yet it was well to have learned. The last thing to be learned and yet one that is most important, is a true sense of perspective. By that I mean the ability to give to things their relative value. It is the lack of this quality that will cause some of you to suffer from gastric ulcer, pernicious anemia or appendicitis following your first lectures on these subjects. Had I realized the importance of certain subjects whose value I failed to appreciate while an undergraduate, I should have saved considerable time and expense during my postgraduate study; and I doubt if I am alone in this regard.

Remember that you are to be first of all, doctors; that if in later years you find that certain lines of work are uncongenial to you and that you, therefore, are better fitted for them than for others, you must have a solid foundation in general medicine upon which to base your special work. No undergraduate school can make specialists and no one can make a greater mistake than to try and start to make himself one while here.

There is another tendency against which I also wish to add my word of warning, and that is the placing of the examinations as a goal. This is apt to be carried over from high school life. Nowhere is such an attitude more to be deplored than in the study of medicine. A grammar school boy whom I once knew, when asked the capital of Illinois answered with a perfectly satisfied air and one not quite free from pride, "I don't know. I don't have to know that now. I passed geography last term." The studies you are to pursue here are to be your stock in trade. You are studying here to gain and retain knowledge which will be of daily use to you in your life work. It is unfortunate that owing to the number of students and the necessarily limited personal contact between teacher and student, examinations are necessary.

The man who loafs during term and then by cramming passes his examinations, as some few may do, may get his degree of M.D. and his legal right to practise at the profession, but success will never be his. No man who does his daily work faithfully need have any fear of examination time, while no man who fails to do it can have anything else. No doubt there are periods of discouragement ahead of you. There are subjects to be mastered as elusive as the proverbial flea. You think you have learned them well, and after a time try to refer back to them but find your mind a blank. But you need not be dismayed. One must get in order to forget, and if you get often enough you cannot forget. I think one of the most helpful things that was said to me on the eve of my medical study, was " Now don't be discouraged with anatomy; you will study it and think you have learned it and then find you have forgotten it. But remember this fact: no one ever knew his anatomy who had not learned it and forgotten it seven times;" and although there does not seem to be sufficient authority for the oft repeated statement that Sir Henry Gray failed in anatomy several times, before he made it his business to so learn it that his text book has been our standard ever since, yet it may well have been true.

Gentlemen, in the name of those for whom I am commissioned and privileged to speak, I welcome you to this school. May you realise the interest your teachers have in your welfare; may you feel that they stand ever ready to assist you not alone as teachers but as friends; and may you, by faithful devotion to your work, so fit yourselves for your profession that they will be pleased to point to you as doctors of their making!

152 ALLEN STREET.

